# Variability of relationship between inner-retinal structural changes and visual dysfunction in optic neuropathy

**DOI:** 10.1038/s41598-024-62704-w

**Published:** 2024-05-27

**Authors:** Hye Jun Joo, Yeji Moon, Jae Ho Jung

**Affiliations:** 1 Department of Ophthalmology, Seoul National University Hospital, Seoul, South Korea; 2 Department of Ophthalmology, Asan Medical Center, University of Ulsan College of Medicine, Seoul, South Korea; 3 Department of Ophthalmology, Seoul National University College of Medicine, 101, Daehak-ro Jongno-gu, Seoul, 03080 South Korea

**Keywords:** Eye diseases, Visual system

## Abstract

Optical coherence tomography (OCT) displays the retinal nerve fiber layer (RNFL) or macular ganglion cell and inner plexiform layer (GCIPL) thickness below 1st percentile in red color. This finding generally indicates severe inner-retinal structural changes and suggests poor visual function. Nevertheless, some individuals show preserved visual function despite these circumstances. This study aimed to identify the correlation between best-corrected visual acuity (BCVA) and inner-retinal thickness based on OCT parameters in various optic neuropathy patients with extremely low RNFL/GCIPL thickness, and determine the limitation of OCT for predicting visual function in these patients. 131 patients were included in the study. The mean BCVA in logMAR was 0.55 ± 0.70 with a broad range from − 0.18 to 3.00. Among the OCT parameters, temporal GCIPL (r = − 0.412) and average GCIPL (r = − 0.366) exhibited the higher correlations with BCVA. Etiological comparisons of optic neuropathies revealed significantly lower BCVA in LHON (all *p* < 0.05). Idiopathic optic neuritis (ON) and MOGAD exhibited better and narrower BCVA distributions compared to the other optic neuropathies. OCT had limited utility in reflecting BCVA, notwithstanding significant inner-retinal thinning after optic nerve injuries. Caution is needed in interpreting OCT findings, especially as they relate to the etiology of optic neuropathy.

## Introduction

Optical coherence tomography (OCT) is a non-invasive, widely utilized diagnostic tool for detecting optic neuropathy and monitoring its progression^1^. Overall diagnostic accuracy has improved with the introduction of spectral-domain OCT (SD-OCT)^2,3^. The advantage of OCT lies in its utility for objective detection of structural changes in the optic disc or retina and prediction of correlations between structural changes (neuronal loss) and visual dysfunction^4^.

Retinal ganglion cell (RGC) elements exist in 3 layers in the retina: the retinal nerve fiber layer (RNFL) containing the ganglion cell axons, the ganglion cell layer consisting of the ganglion cell bodies, and the inner plexiform layer comprising the ganglion cell dendrites. For OCT analysis, the last two layers are designated the ganglion cell–inner plexiform layer (GCIPL)^5^. After an optic neuropathy attack, thinning of the RNFL and GCIPL occurs. RNFL thinning on OCT typically occurs approximately 2–6 months after the acute episode, with subsequent stabilization observed around 7–12 months^6,7^. Recent studies have reported that GCIPL thinning can precede RNFL thinning in various optic neuropathies, with damage to axons resulting in RGC death within days to weeks, while RNFL thinning occurs within 1–2 months^8,9^. In this context, not only RNFL thickness analysis but also retinal GCIPL analysis has become very important clinically in evaluation of various optic neuropathies including glaucomatous optic neuropathy.

When RNFL and/or GCIPL thickness on OCT drops below the 1st percentile relative to the normal population, the OCT program automatically displays them in red color, and the patient is categorized as “(being) in the red zone.” This finding generally indicates severe inner-retinal structural changes and suggests poor visual function. Nevertheless, some individuals show preserved visual function despite these circumstances.

We therefore investigated the relationship between GCIPL/RNFL thickness and visual function in various optic neuropathy patients showing significant thinning of both the RNFL and GCIPL (i.e., in the red zone).

## Methods

### Study design and subjects

This retrospective cohort study included 131 patients aged 18 years or more who had been diagnosed with optic neuropathy between 2017 and 2023 at the Seoul National University Hospital Neuro-ophthalmology Clinic. Patients with a history of demyelinating optic neuritis (ON), non-arteritic ischemic optic neuropathy (NAION), traumatic optic neuropathy, compressive optic neuropathy, toxic optic neuropathy or Leber's hereditary optic neuropathy (LHON) were included. ON was classified based on serological antibody status. Therefore, we included only patients who had been tested for the representative biomarkers of ON, namely MOG-IgG and AQP4-IgG. Serum samples were obtained at the time of an acute attack and tested for the presence of AQP4-IgG and MOG-IgG. Testing for AQP4-IgG and MOG-IgG was conducted using a live-cell-based assay. The ON patients were classified into three disease groups according to the disease etiology, based on the following criteria: idiopathic ON group: patients who were seronegative for both AQP4 and MOG IgG, and did not meet the diagnostic criteria for multiple sclerosis (MS); myelin oligodendrocyte glycoprotein antibody-associated disease (MOGAD) group: patients who were seronegative for AQP4-IgG and seropositive for MOG-IgG; neuromyelitis optica spectrum disorder (NMOSD) group: patients who were seropositive for AQP4-IgG and seronegative for MOG-IgG.

To eliminate the effect of acute optic neuropathy, we included only patients who had been diagnosed with optic neuropathy more than one year previously. In addition, we defined non-progression as less than 2% of deterioration over the course of more than 6 months relative to the previous OCT data^10,11^. All of the included patients showed average GCIPL and RNFL thicknesses below the 1st percentile on OCT (red zone).

The exclusion criteria were as follows: (1) progression in the mean OCT value of 2% or more during follow-up, (2) poor OCT scans (signal strength < 6 or motion artifacts), (3) presense of any other ocular disorders that could interfere with OCT image acquisition such as severe cataract, ocular disorders which can affect quality of OCT scans or RNFL/GCIPL measurements, such as glaucoma, or any macular disease such as AMD and ERM that could interfere with segmentation of retinal layers, (4) refractive errors >  + 3D or < − 6D, (5) history of neurological disorders or concurrent central nervous system lesions in patients with ON, (6) cases in which two neuro-ophthalmologists (JHJ, Y-M) disagreed on the diagnosis.

The data on peripapillary RNFL and macular GCIPL thickness were collected using spectral-domain OCT (Cirrus HD SD-OCT, Carl Zeiss Meditec, Inc., Dublin, CA, USA). The mean macular thickness (from ILM to RPE) was assessed based on a 6 mm × 6 mm data cube, and the central macular thickness represented the mean thickness in the central 1000-μm diameter area. Images of signal strength ≥ 6 were accepted, and layer-by-layer segmentation was conducted by instrument-embedded software. Following this initial step, we assessed the B-scan images and checked for adequacy, and segmentation errors were modified manually by two independent neuro-ophthalmology specialists (Y-M and JHJ) in a double-blind procedure. In cases where discrepancies existed between the two examiners, we excluded them from the analysis. A GCIPL sector analysis was conducted for four zones: superior, inferior, temporal, and nasal. The temporal sector parameter result was obtained by averaging the supero- and inferotemporal zones, and the nasal sector was obtained by averaging the supero- and inferonasal zones.

We collected data on best-corrected visual acuity (BCVA) and converted it to logMAR for analysis. Poor vision measured by means of count finger (CF), hand motion (HM), light perception (LP), and no light perception (NLP) was converted to logMAR 1.7, 2.0, 2.3, and 3.0, respectively^12^. Patients capable of undergoing the Humphrey Visual Field Analyzer (Carl Zeiss Meditec, Dublin, CA, USA) underwent the examination.

This study was performed in accordance with the tenets of the Declaration of Helsinki and approved by the Institutional Review Board (IRB) of Seoul National University Hospital (IRB no. 2307-128-1450). The need to obtain informed consent was waived because of the retrospective study design and use of anonymized clinical data.

### Analysis

Kolmogorov–Smirnov test was used to assess normal distribution in continuous variables. To examine the differences between two independent groups of continuous variables, the Mann–Whitney U test was conducted. Additionally, to assess the linear relationship between two continuous variables, the Spearman correlation analysis was performed. Except where stated otherwise, the data herein are presented as mean ± SD, the level of statistical significance having been set at a two-sided *p* value of < 0.05. The value of Snellen visual acuity was transformed to logarithmic units. For patients with bilateral optic neuropathy, the eye with the worse RNFL/GCIPL value was used for the analysis. All of the statistical analyses were performed using SPSS version 27.0 (IBM, Armonk, NY, USA).

## Results

**Figure 1:**
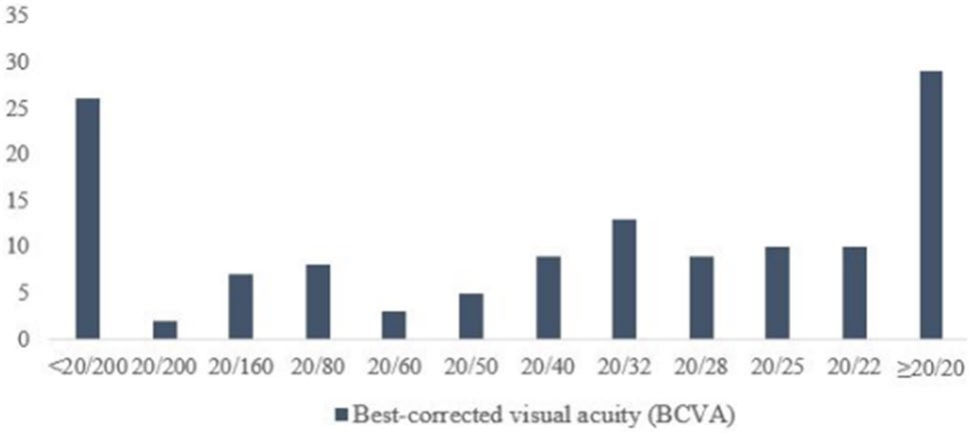
Overall distribution of best-corrected visual acuity (BCVA) in patients with retinal nerve fiber layer (RNFL) and ganglion cell and inner plexiform layer (GCIPL) thickness below 1% (red zone) relative to normal population. The overall distribution of BCVA showed a broad range.

A total of 131 eyes of 131 patients were enrolled in this study. The baseline demographic data are as follows: 46 males (35.1%) and 85 females (64.9%), with an average age at examination of 54.82 ± 13.84 [18–79] years. The mean spherical equivalent (SE) was − 1.27 ± 2.14 [− 6.00 to + 3.00]. The mean BCVA in logMAR was 0.55 ± 0.70 [− 0.18 to 3.00], showing a broad range. Approximately 20% of the patients exhibited BCVA of ≥ 20/20 or below 20/200 (Fig. 1).

**Figure 2:**
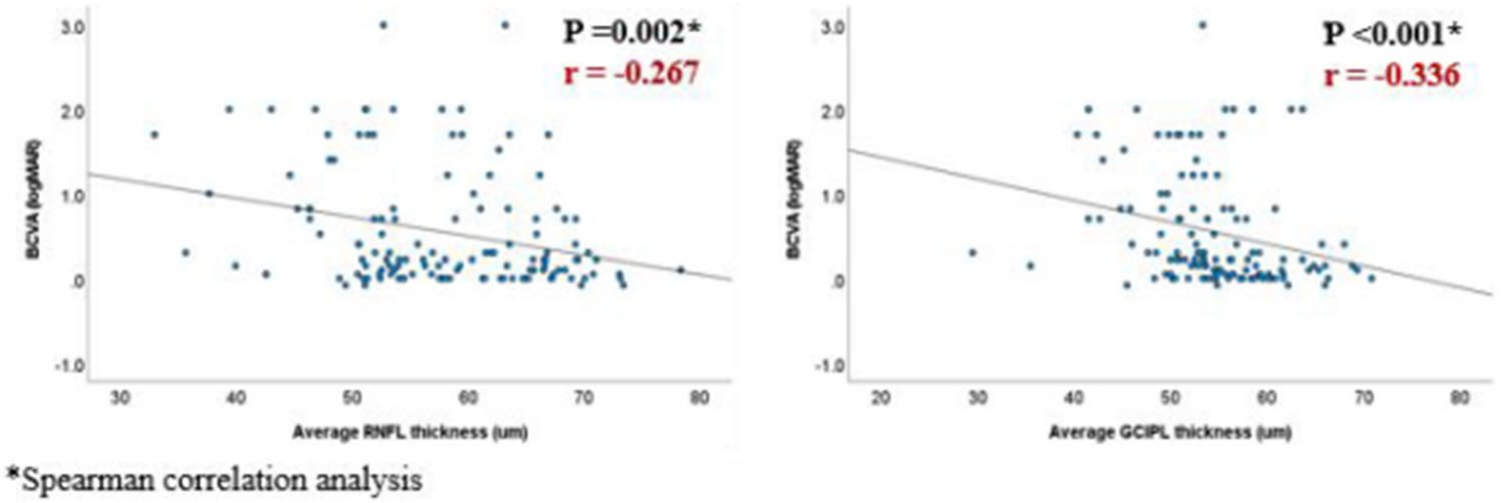
Scatter plots showing the correlation between best-corrected visual acuity (BCVA) and average retinal nerve fiber layer (RNFL)/ganglion cell and inner plexiform layer (GCIPL) thickness. The spearman correlation analysis between BCVA and average RNFL/GCIPL thickness showed a statistically significant (*p* = 0.002 and *p* < 0.001, respectively), but low correlation coefficient (r = − 0.267 and r = − 0.366, respectively).

The correlations between average RNFL and GCIPL thickness and BCVA, respectively, were investigated. Statistical significance was shown (*p* = 0.002 and *p* < 0.001, respectively); however, the strength was relatively low in both cases (r = − 0.267 and r = − 0.366, respectively) (Fig. 2). When analyzed by sector on OCT, temporal GCIPL (r = − 0.412) and average GCIPL (r = − 0.366), among the OCT parameters, exhibited relatively higher correlations with BCVA (Table 1). Moreover, the analysis involving patients able to undergo quantitative visual field (VF) test showed that the mean deviation of VF result displayed a moderate correlation with RNFL parameters, notably average RNFL thickness (r = 0.605) (Table 1).

**Table 1:** Correlations between optical coherence tomography (OCT) sector parameters and best-corrected visual acuity (BCVA)/mean deviation of visual field (MD of VF).

	BCVA (n = 131)		MD of VF (n = 88)	
	Rho	*p*-value*	Rho	*p*-value*
Macular thickness (um)				
Central thickness	0.015	0.864	− 0.121	0.263
Mean thickness	**− 0.269**	**0.002**	**0.378**	** < 0.001**
RNFL parameter (um)				
Average	**− 0.267**	**0.002**	**0.605**	** < 0.001**
Superior	**− 0.251**	**0.004**	**0.572**	** < 0.001**
Temporal	0.157	0.073	− 0.080	0.461
Inferior	**− 0.353**	** < 0.001**	**0.562**	** < 0.001**
Nasal	0.021	0.814	0.082	0.447
GCIPL parameter (um)				
Average	**− 0.366**	** < 0.001**	**0.313**	**0.003**
Superior	**− 0.318**	** < 0.001**	**0.175**	0.102
Temporal	**− 0.412**	** < 0.001**	**0.397**	** < 0.001**
Inferior	**− 0.247**	**0.004**	**0.274**	**0.010**
Nasal	**− 0.227**	**0.009**	**0.158**	0.140

Significant values are in [bold].

BCVA best-corrected visual acuity, MD mean deviation, VF visual field, RNFL retinal nerve fiber layer, GCIPL ganglion cell and inner plexiform layer.

*Spearman correlation analysis.

### Subgroup analysis based on etiologies of optic neuropathies

**Figure 3:**
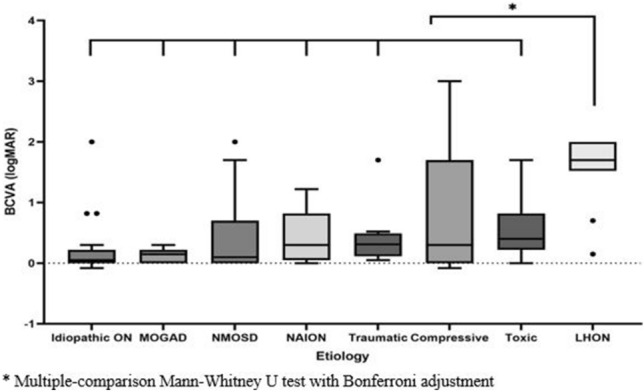
Multiple-comparison Mann–Whitney U test with Bonferroni adjustment showed the best-corrected visual acuity (BCVA) distribution based on the etiologies of optic neuropathies. The BCVA of LHON patients was significantly lower than that of the other etiologies (all *p* < 0.05). Box-and-whisker plot showed that idiopathic optic neuritis (ON) and myelin oligodendrocyte glycoprotein antibody-associated disease (MOGAD) were associated with relatively good BCVA and had a narrow distribution range, relative to the other optic neuropathies. The lower quartiles of toxic optic neuropathy and Leber's hereditary optic neuropathy (LHON) were located above the upper quartiles of idiopathic ON and MOGAD.

The BCVA and average RNFL/GCIPL thicknesses according to etiologies are presented in Table 2. The BCVA of LHON patients was significantly lower than that of the other etiologies (idiopathic ON, MOGAD, NMOSD, NAION, traumatic optic neuropathy, and toxic optic neuropathy) (Fig. 3, all *p* < 0.05). Additionally, the box-and-whisker plot in Fig. 3 showed that idiopathic ON and MOGAD were associated with relatively good BCVA and had a narrow distribution range, relative to the other optic neuropathies. Compressive optic neuropathy showed a wide BCVA distribution range. Meanwhile, the lower quartiles of toxic optic neuropathy and LHON were located above the upper quartiles of idiopathic ON and MOGAD.

**Table 2:** Best-corrected visual acuity (BCVA), retinal nerve fiber layer (RNFL) thickness and ganglion cell and inner plexiform layer (GCIPL) thickness based on etiologies.

Etiology	BCVA (log MAR)	RNFL thickness (um)	GCIPL thickness (um)
Total (n = 131)	0.55 ± 0.70	57.82 ± 8.80	54.53 ± 6.80
Idiopathic ON (n = 23)	0.21 ± 0.46	60.13 ± 9.27	56.09 ± 6.18
MOGAD (n = 11)	0.13 ± 0.12	57.33 ± 4.33	55.86 ± 4.53
NMOSD (n = 21)	0.41 ± 0.61	55.48 ± 12.10	52.51 ± 8.47
NAION (n = 15)	0.44 ± 0.40	53.08 ± 7.20	55.24 ± 7.50
Traumatic optic neuropathy (n = 8)	0.44 ± 0.53	50.71 ± 5.12	54.27 ± 9.22
Compressive optic neuropathy (n = 19)	0.82 ± 1.03	60.37 ± 7.10	56.96 ± 6.70
Toxic optic neuropathy (n = 2)	0.60 ± 0.53	62.54 ± 7.10	53.30 ± 5.50
LHON (n = 11)	1.56 ± 0.60	55.35 ± 6.73	51.35 ± 4.88

*BCVA* best-corrected visual acuity, *RNFL* retinal nerve fiber layer, *GCIPL* ganglion cell and inner plexiform layer, *MOGAD* Myelin oligodendrocyte glycoprotein antibody-associated disease, *NMOSD* Neuromyelitis optica spectrum disorder, *NAION* Non-Arteritic Anterior Ischemic Optic Neuropathy, *LHON* Leber hereditary optic neuropathy.

Values are presented as mean ± standard deviations.

## Discussion

Our study revealed a broad range of visual acuity in extremely low RNFL/GCIPL (red zone) patients. The RNFL and GCIPL thickness values showed a weak correlation with visual acuity, the GCIPL thickness values showing a relatively better correlation with BCVA than with RNFL thickness. Even in cases displaying similarly extreme thinning on OCT, we observed a tendency for varying BCVA distributions based on the underlying cause of the optic neuropathy. LHON exhibited a lower visual acuity distribution, while MOGAD and idiopathic ON showed favorable visual acuity distributions, even with significant RNFL and GCIPL thinning.

Previous studies on various types of optic neuropathies revealed a correlation between structure and visual function, and proposed optimal cutoff values of RNFL and GCIPL thickness for prediction of favorable visual outcomes in NAION: RNFL thickness > 61.5 um or macular GCIPL thickness > 52.5 um for BCVA 20/40 or better^13^, and RNFL thickness > 75 um as the threshold for visual recovery after ON^7^. However, our study could not specify the OCT cut-off value for favorable visual function. Additionally, and perhaps significantly, the current study showed various structure–function relationships, indicating that structural changes may not adequately reflect visual function and can vary among etiologies.

A previous study by Kim et al. reported a limited correlation between SD-OCT parameters and BCVA in open-angle glaucoma patients, which was indicative of a wide variability of the structure–visual acuity relationship in glaucoma. They concluded that as central visual acuity can be influenced by various factors, it is not possible to estimate it from OCT-measured RNFL or GCC thickness alone^14^. The results of our study were comparable, showing divergent visual acuity distributions and a low correlation between visual acuity and GCIPL/RNFL thickness. We observed a better correlation, though still not a high one, between GCIPL thickness and visual acuity than for RNFL thickness. This finding is consistent with the study by Rebolleda et al.^15^, wherein they observed a strong correlation between BCVA and papillomacular bundle in NAION eyes. RNFL thickness as measured by commercially available OCT is determined not just by nerve fibers but also by non-neuronal (e.g., glial) tissues^16^. We could hypothesize that the reactive glial tissue proliferation following axonal damage may contribute to this outcome. Consequently, RNFL thickness on OCT examination may not accurately represent the actual count of RGCs, leading to a reduced correlation between RNFL thickness and visual function. Moreover, a previous study reported that the macular GCIPL parameters represent more favorable indicators than do RNFL parameters for monitoring of compressive optic neuropathy, which fact was attributed to the notably reduced variance (standard deviation) observed in normal healthy eyes^17^. Indeed, our study showed that the structure–function correlation is not always consistent in optic neuropathies. New methods for distinguishing between neural and glial tissues during RNFL measurements may lead to better correlations between OCT parameters and visual function in the near future.

The reasons for the broad visual acuity distribution according to specific diseases may not be clear, though tentative hypotheses can be made. Among the various conditions exhibiting similarly extremely low RNFL and GCIPL thickness, LHON was associated with notably worse visual impairment. The reason that LHON patients show a worse visual prognosis within the same red zone can be attributed to the limitation of assessing structural changes through OCT, which is due to a floor effect caused by glial tissue or blood vessels^18^. This could limit assessment of structural changes by OCT in cases of extremely thin RNFL and/or GCIPL thickness. Moreover, the worse visual prognosis in LHON could be attributed to the involvement of the papillomacular bundle, given the nature of the disease^19^.

The present box-and-whisker-plot-based analysis indicated that toxic optic neuropathy and LHON exhibited a worse visual prognosis relative to idiopathic ON and MOGAD (Fig. 3). Toxins causing optic neuropathies are thought to impair metabolism, and mitochondrial oxidative phosphorylation^20^. This mitochondrial dysfunction results in the death of RGCs, ultimately causing visual dysfunction. In turn, damage to or death of RGCs leads to inherited optic neuropathy^19^. On the other hand, the acute inflammatory process in ON causes significant axonal loss, which eventually results in RGC neuronal loss through retrograde degeneration^21,22^. It may be suggested that the primary target tissue affected may influence visual outcomes among the various optic neuropathies.

The findings of our study showed that compressive optic neuropathy cases within the red zone exhibit relatively maintained RNFL and GCIPL thickness, with notable variability in BCVA distribution. A previous study on the OCT scans of newly diagnosed compressive optic neuropathy patients showed similar results, indicating various forms of GCIPL changes^17^. Moreover, the same study’s structure–function analysis of compressive optic neuropathy with comparable thinning profiles revealed a spectrum, rather than a fixed value, of visual-field loss associated with each thinning profile. As compressive optic neuropathy generally occurs over a gradual and chronic course, a delay in structural thinning relative to functional damage may exist^17^. Even if OCT measurements indicate similar thickness, variations among the components contributing to thickness may arise due to differing disease progressions. This may help explain why, despite similar thinning of RNFL and GCIPL thicknesses, distinct visual functions can be shown.

There are some limitations to this study. First, for patients with vision of 20/100 or lower, quantitative automated visual-field testing could not be conducted, due to poor cooperativeness. Therefore, the evaluation of visual function was based on visual acuity, which is only a partial measure of visual impairment and does not perfectly reflect visual function. Second, the small sample size restricted within-group comparison. For example, we could not analyze the cases of patients with MS, due to the small number of patients who had thinning of both layers without CNS lesions. Third and finally, the study was conducted using only one type of equipment, indicating a potential limitation in terms of generalizability or comprehensive assessment. A future prospective study with a larger cohort would provide more information on the structure–function relationship in optic neuropathies.

In conclusion, our results demonstrate that the OCT values for patients with extremely low RNFL and GCIPL thickness cannot fully represent visual function. Considering the wide variability of the structure–visual acuity relationship, clinicians should take other variables into account for prediction of visual acuity in patients with various optic neuropathies.

## Data Availability

The datasets generated and/or analysed during the current study are not publicly available due to the information security policy of the hospital but are available from the corresponding author on reasonable request.
